# Effect of postpartum calcium supplementation on serum calcium and parathyroid hormone concentrations in multiparous Holstein cows

**DOI:** 10.3168/jdsc.2023-0455

**Published:** 2023-12-09

**Authors:** I.R. Frost, C.R. Seely, H.A. McCray, K.R. Callero, J.A. Seminara, R.M. Martinez, A.M. Reid, C.N. Wilbur, K.J. Koebel, J.A.A. McArt

**Affiliations:** 1 College of Agriculture and Life Sciences, Cornell University, Ithaca, NY 14853; 2 Department of Population Medicine and Diagnostic Sciences, College of Veterinary Medicine, Cornell University, Ithaca, NY 14853; 3 College of Veterinary Medicine, Cornell University, Ithaca, NY 14853; 4 College of Arts and Sciences, Cornell University, Ithaca, NY 14853

## Abstract

Although postpartum Ca supplementation strategies are often employed to prevent subclinical hypocalcemia in dairy cows, these strategies have produced a mix of beneficial, neutral, and detrimental results when assessing milk yield and subsequent disease outcomes. Because the mechanisms underlying these differing results are unknown, our objectives were to determine how common postpartum Ca supplementation strategies affect blood Ca concentrations and parathyroid hormone (PTH). We conducted a randomized controlled trial with 74 multiparous dairy cows on a commercial dairy in central New York. Cows were assigned to 1 of 4 supplementation groups immediately after calving: (1) control (CON; no Ca supplementation, n = 15); (2) conventional oral Ca supplementation (BOL-C; 43 g of oral Ca bolus administered immediately after calving and 24 h later, n = 17); (3) delayed oral Ca supplementation (BOL-D; 43 g of oral Ca bolus administered 48 and 72 h after calving, n = 15); or (4) subcutaneous infusion (SQ; 500 mL of 23% Ca borogluconate infused subcutaneously once immediately after calving, n = 15). Blood samples were collected immediately after calving (0 h) and at 8, 16, 24, 32, 40, 48, 56, 64, 72, 80, 88, 96, 120, and 168 h postpartum for a total of 15 blood samples per cow. Cows were excluded if administered Ca, via any route, by farm employees or if they died or were sold within 96 h following parturition, which left 62 cows for analysis. Linear mixed models, accounting for repeated measures, were created to analyze changes in serum total Ca (tCa) and PTH over the first 168 h after parturition and assess differences between supplementation groups. Serum tCa and PTH concentrations were not different at the time of calving among supplementation groups. There was a supplementation group by hour postcalving interaction for mean tCa concentration in which SQ cows had reduced tCa concentrations from 32 to 64 h compared with CON cows, 32 to 96 h compared with BOL-C cows, and 40 to 64 h compared with BOL-D cows. Mean PTH concentration did not differ among supplementation groups across 168 h after enrollment and was 158.1 pmol/L (95% confidence interval [CI] = 148.2 to 168.0) for CON cows, 164.0 pmol/L (95% CI = 154.9 to 173.1) for BOL-C cows, 158.7 pmol/L (95% CI = 149.2 to 168.1) for BOL-D cows, and 153.2 pmol/L (95% CI = 143.6 to 162.8) for SQ cows. Our findings suggest that although serum tCa does not differ between cows that receive conventional or delayed oral Ca bolus supplementation at calving and cows that receive no supplemental Ca, subcutaneous infusion of Ca at calving reduces serum tCa for a substantial period between 32 and 64 h postsupplementation. However, as PTH concentrations did not differ among groups across 168 h postpartum, the mechanism by which tCa is reduced remains unclear.

## Graphical Abstract

**Figure:**
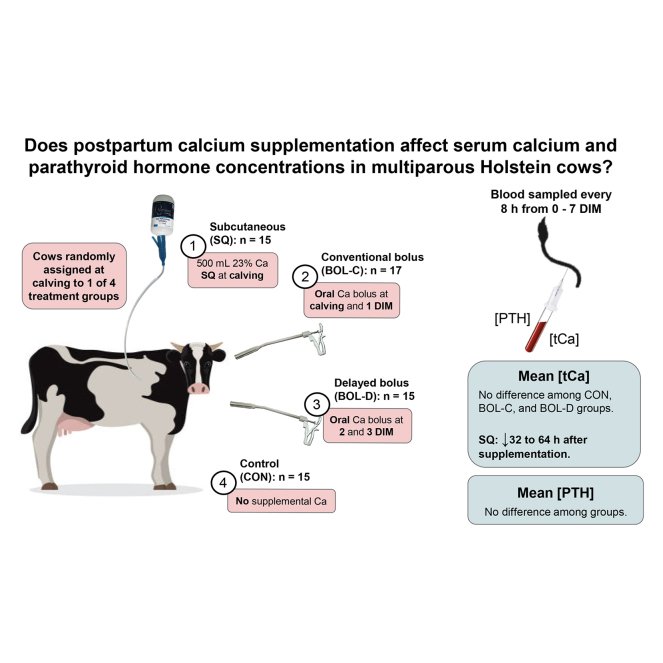

Postparturient hypocalcemia in dairy cows is a common occurrence that results from the onset of lactation and an inability to immediately compensate for the heightened Ca demand. An estimated 45% of multiparous dairy cows will experience some form of subclinical hypocalcemia (Reinhardt et al., 2011; defined as total Ca (**tCa**) ≤2.0 mmol/L within 48 h postpartum), and as such, postpartum subclinical hypocalcemia preventive approaches, including oral Ca supplementation and subcutaneous infusion of Ca, are routinely used on 69% of US dairy farms (USDA-NAHMS, 2014). However, beneficial responses to such supplementations vary based on the presence of an increase in milk yield or a decrease in disease events.

It remains unknown why postpartum Ca supplementation, although it increases blood Ca concentration (Blanc et al., 2014; Domino et al., 2017), has minimal effect on economically important outcomes when administered in a blanket method to all cows (Valldecabres et al., 2023). It is similarly unclear why some postparturient multiparous cows positively benefit from receiving oral Ca boluses whereas others show no benefit, and why multiparous cows that receive subcutaneous Ca experience no subsequent reduction in disease events or increase in milk yield (Miltenburg et al., 2016; Domino et al., 2017).

Parathyroid hormone (**PTH**), a primary regulator of Ca homeostasis, has been found to peak within 24 h postcalving and decrease through 48 to 72 h postpartum (Hernández-Castellano et al., 2017; Vieira-Neto et al., 2017). In both studies, administration of substances hypothesized to sustain blood Ca concentration (5-hydroxy-l-tryptophan, Hernández-Castellano et al., 2017, administered before calving; calcitriol, Vieira-Neto et al., 2017, administered within 6 h postpartum) removed the peak of PTH postcalving. Administration of intravenous Ca to recumbent cows with clinical hypocalcemia also causes a reduction in PTH, as blood Ca rises to a concentration that reduces the secretion of PTH (Braun et al., 2009). It is possible that administration of exogenous substances, including routine Ca supplementation at the time of calving, impedes homeostatic Ca regulation via PTH in some cows, subsequently reducing the expected benefit of increased blood Ca concentrations. Thus, our objectives were to determine if postpartum Ca supplementation strategies affect blood PTH and Ca concentrations across the first 7 DIM. Our hypothesis was that infusion of subcutaneous Ca immediately after calving would cause a greater reduction in PTH concentrations than that resulting from conventional or delayed oral bolus administration when compared with cows not supplemented with Ca.

All cow procedures were reviewed and approved by the Cornell University Institutional Animal Care and Use Committee, protocol number 2020–0040. We conducted a randomized controlled trial and have described our findings following REFLECT reporting guidelines (O'Connor et al., 2010). We calculated our sample size based on our primary outcome of interest: a difference in serum PTH concentration 96 h after parturition between each supplementation group and the control group. Assuming a difference in mean serum PTH concentration of 35 pmol/L between groups, and a group-level standard deviation of 25 pmol/L (Hernández-Castellano et al., 2017; 1 ng/mL = 106 pmol/L) while controlling for a 5% type I error with 80% power, we needed to enroll 16 cows per group (total n = 64). To account for loss to follow-up or study exclusion, we increased our sample size by 10% and aimed to enroll 72 cows.

We conducted our study in July 2021 on a commercial dairy in central New York milking approximately 4,400 Holstein cows. This farm was selected based on its long-term relationship with the Cornell Ambulatory and Production Medicine Clinic and its willingness to participate in research trials. During the close-up dry period, cows were fed a negative DCAD TMR, formulated at −13.0 mEq/100 g of DM, once daily at 1100 h and were monitored every 30 min by trained farm employees for signs of parturition at which time they were moved to individual maternity pens using a “just-in-time” calving strategy. Cows were milked near the maternity pen within 8 h following parturition and then moved to a freestall fresh pen where they remained for the duration of the study. Fresh cows were milked 3 times a day in a 100-stall rotary parlor and fed an early-lactation TMR once daily at 0745 h.

Our research team monitored the individual maternity pens for parturition 24 h per d during the enrollment period, and multiparous cows were assigned to supplementation group immediately after parturition following a randomized block design. Within each of the 18 blocks, cows were randomly assigned to 1 of 4 supplementation groups using the randomization function in Excel (Microsoft). Cows were sequentially allocated, independent of parity, based on the randomized list to supplementation group: (1) control (**CON**, n = 18), no postpartum Ca supplementation; (2) conventional oral Ca supplementation (**BOL-C**, n = 19), 43 g of oral Ca bolus (Bovikalc, Boehringer Ingelheim Animal Health) immediately after calving and a second 43 g of oral Ca bolus 24 h later; (3) delayed oral Ca supplementation (**BOL-D**, n = 19), a 43 g of oral Ca bolus 48 and 72 h after calving; or (4) subcutaneous Ca (**SQ**, n = 18), one subcutaneous infusion of 500 mL of 23% Ca borogluconate (10.7 g of Ca; Radix Labs) immediately following calving in a single location caudal to the shoulder over a period of 5 min. Initial supplementations were administered immediately after calving within the maternity pens, and supplementations between 24 and 72 h were administered in a sort-gate rail with a headlock following milking. Assignment to supplementation group, Ca administration, and blood sampling were conducted by the research team, and all Ca supplementations were administered after blood sample collection for each designated time point described below. Farm employees were blinded to cow supplementation-group assignment.

Blood samples were collected immediately after calving (0 h) and 8, 16, 24, 32, 40, 48, 56, 64, 72, 80, 88, 96, 120, and 168 h postpartum for a total of 15 blood samples per cow. The 0-h samples were collected in the maternity pen, and all subsequent samples were collected upon exit from the milking parlor at 0230, 1030, and 1830 h. Thus, depending on the time of calving relative to milking of the fresh pen, the 8-h sample occurred between 4 to 12 h postpartum with all following sample time points occurring in 8-h measurements. Blood samples were collected from the coccygeal vessels into a 10-mL Vacutainer tube containing no anticoagulant (Becton Dickinson) and immediately placed on ice. Blood samples were transported to Cornell University within 4 h of collection and centrifuged at 2,000 × *g* for 20 min at 4°C. Serum was harvested, aliquoted into 1.7-mL microfuge tubes, and stored at −20°C until analysis.

Serum samples were analyzed for tCa using commercially available kits (Calcium Gen. 2, Roche Diagnostics) at the New York State Animal Health and Diagnostic Center on an automated analyzer (Hitachi Modular P800, Roche Diagnostics). Inter- and intra-assay coefficients of variation were 1.1% and 0.9%, respectively. Serum samples were analyzed for PTH by the first author (IRF) using Bovine Intact PTH ELISA kits (DEIA1826B, Creative Diagnostics) following the manufacturer's instructions as previously validated by Connelly et al. (2021). Inter- and intra-assay coefficients of variation were 13.0% and 2.9%, respectively.

Enrolled cows were excluded from analysis if administered Ca, via any route, by farm employees or if they died or were sold within 96 h following parturition. Cows administered Ca by farm employees >96 h following parturition were retained in the study, but their individual time point data were excluded from analysis following Ca administration. All analyses were conducted using SAS v.9.4 (SAS Institute Inc.).

Differences between baseline tCa and PTH among supplementation groups were assessed via univariable analysis using the ANOVA procedure. Residuals were visually assessed for normality. Differences in parity group (2, ≥3) between supplementation groups were assessed through the FREQ procedure with a univariable analysis via Fisher's exact test.

Changes in tCa and PTH over time and differences between supplementation groups were analyzed via generalized linear mixed models created using the MIXED procedure. Multiple measurements over time within the same cow were accounted for using hours postcalving in the REPEATED statement. Models included the random effect of cow within supplementation group and the fixed effects of supplementation group, parity group, hours postcalving, 0 h tCa, or 0 h PTH concentration, and the interactions of supplementation group by hours postcalving and supplementation group by parity group. Regardless of statistical significance, baseline 0 h tCa and 0 h PTH remained in their respective models to account for upper and lower concentration boundaries. The interactions supplementation group by parity group was removed via stepwise backward elimination if *P* > 0.10. Given our objectives, the interaction of supplementation group and hours postcalving remained in all models regardless of the level of statistical significance. Several covariance structures were investigated, and unstructured covariance resulted in the best fit model based on the lowest Akaike's information criterion for both the tCa and PTH models. The presence of influential points in each model was assessed using the INFLUENCE statement, and the Cook's D was interpreted for all cows. If the Cook's D was ≥2, then the cow was deemed influential and removed from the analysis; however, no datapoints yielded a Cook's D > 0.13. Residuals were visually assessed for normality. All assumptions for linear regression modeling were met and variable transformations were not required.

When the supplementation group by hours postcalving differed at *P* < 0.05, Bonferroni adjustments were used to account for multiple comparisons. Results are reported as least squares means and their associated 95% confidence intervals. Graphs were designed using Excel.

We enrolled 74 multiparous cows immediately after calving into CON (n = 18), BOL-C (n = 19), BOL-D (n = 19), and SQ (n = 18) supplementation groups. Twelve cows were excluded from analysis (CON, n = 3; BOL-C, n = 2; BOL-D, n = 4; SQ, n = 3) based on Ca administration by farm employees within 96 h following parturition (n = 11) or inability to locate the cow on the farm (n = 1). Of the 11 cows receiving Ca based on farm protocols, 6 were diagnosed with clinical hypocalcemia (CON, n = 2; BOL-D, n = 2; SQ, n = 2), 3 had a difficult calving requiring significant assistance (CON, n = 1; BOL-D, n = 2), 1 was diagnosed with severe clinical mastitis (SQ, n = 1), and 1 had an undefined diagnosis that required supportive care (BOL-C, n = 1). Thus, 62 cows were included in our final analysis (CON, n = 15; BOL-C, n = 17; BOL-D, n = 15; SQ, n = 15). Two cows (CON, n = 1; SQ, n = 1) were diagnosed with severe clinical mastitis at 7 DIM, and thus we excluded their 168-h samples from analysis.

Serum 0 h tCa did not differ among supplementation groups (*P* = 0.63) with group-level mean ± standard deviation concentrations of CON = 2.05 ± 0.18 mmol/L, BOL-C = 2.07 ± 0.22 mmol/L, BOL-D = 2.13 ± 0.18 mmol/L, and SQ = 2.06 ± 0.16 mmol/L. Modeled mean serum tCa concentrations over 168 h after enrollment for each supplementation group are in Table 1, and patterns of tCa over the study period are in Figure 1A. Serum tCa varied over time between supplementation groups (*P* < 0.001) when controlling for 0 h tCa (*P* = 0.22) and parity group (*P* = 0.09). Differences in tCa concentration over time between supplementation groups were especially notable between 32 and 64 h postcalving in which SQ cows had reduced tCa concentrations from 32 to 64 h compared with CON cows, 32 to 96 h compared with BOL-C cows, and 40 to 64 h compared with BOL-D cows (all *P* < 0.10; Figure 1A). Although cows were randomized within block, variations in the time between calving and the first 8-h sampling point might have been unequal among treatment groups. This is a potential limitation of our study design and might have affected mean tCa concentration at the 8-h time point.

**Table 1:** Modeled means and 95% CI of total Ca (tCa) and parathyroid hormone (PTH) of n = 62 multiparous Holstein cows enrolled at calving in a randomized controlled trial and followed for 168 h^1^

Model	Supplementation group				*P*-value					
	CON	BOL-C	BOL-D	SQ	Time	SG	Parity^2^	tCa_0_^3^	PTH_0_^4^	SG × time
tCa, mmol/L	2.19	2.24	2.19	2.08	<0.001	0.005	0.09	0.22	—	<0.001
	(2.12, 2.26)	(2.18, 2.30)	(2.13, 2.26)	(2.01, 2.14)						
PTH, pmol/L	158.1	164.0	158.7	153.2	0.002	0.4	<0.001	—	<0.001	0.3
	(148.2, 168.0)	(154.9, 173.1)	(149.2, 168.1)	(143.6, 162.8)						

1 Cows were assigned to 1 of 4 supplementation groups (SG) immediately after calving: (1) control (CON; no Ca supplementation, n = 15); (2) conventional oral Ca supplementation (BOL-C; 43 g of oral Ca bolus administered immediately after calving and 24 h later, n = 17); (3) delayed oral Ca supplementation (BOL-D; 43 g of oral Ca bolus administered 48 and 72 h after calving, n = 15); or (4) subcutaneous infusion (SQ; 500 mL of 23% Ca borogluconate infused subcutaneously once immediately after calving, n = 15).

2 Parity groups: 2, ≥3.

3 Baseline serum tCa at 0 h postcalving.

4 Baseline serum PTH at 0 h postcalving.

**Figure 1:**
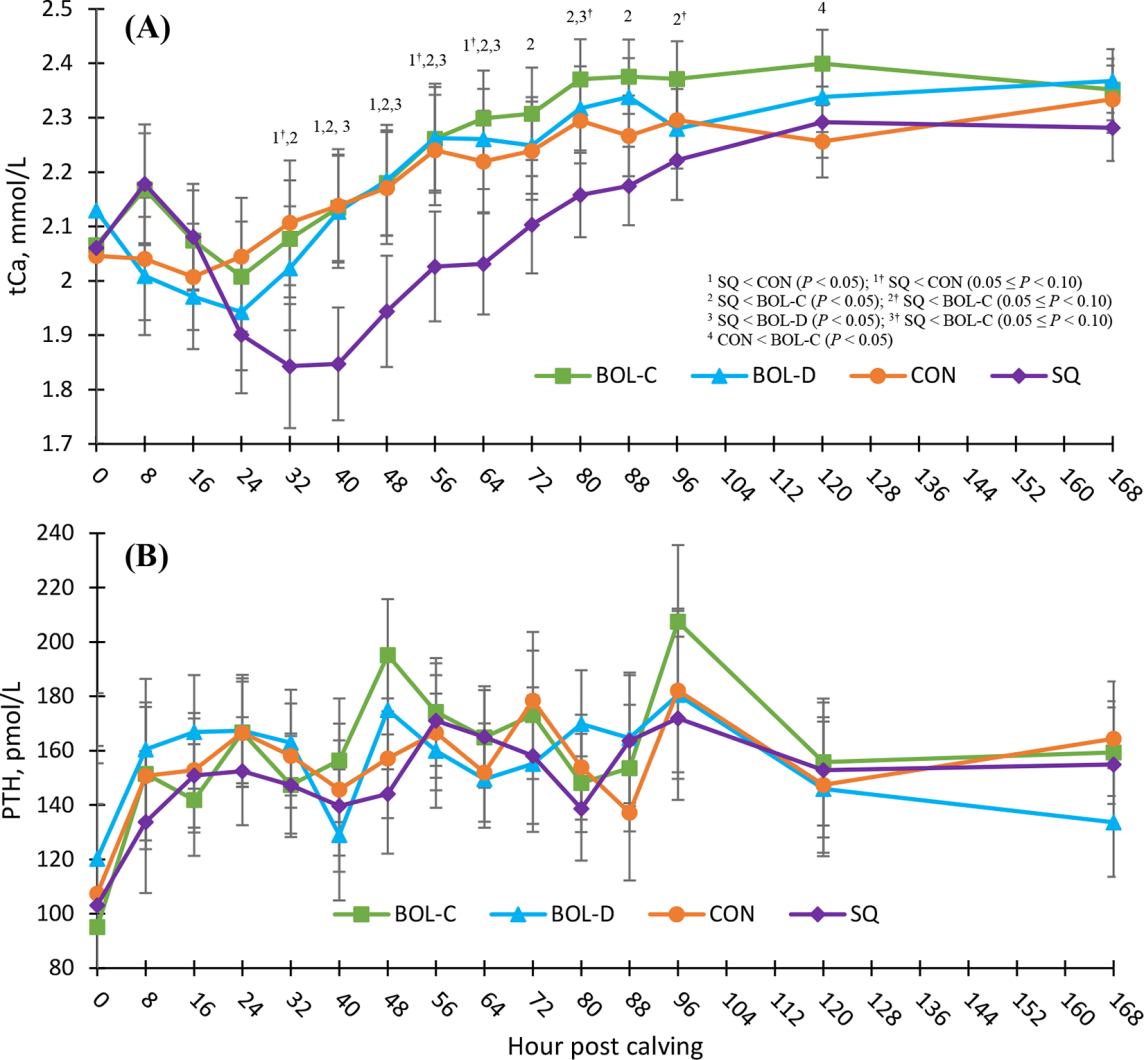
Least squares means and 95% CI of (A) serum total Ca (tCa) and (B) parathyroid hormone (PTH) of n = 62 multiparous Holstein cows enrolled at calving in a randomized controlled trial and followed through 168 h after enrollment. Cows were assigned to 1 of 4 groups immediately after calving: (1) control (CON; no Ca supplementation, n = 15); (2) conventional oral Ca supplementation (BOL-C; 43 g of oral Ca bolus administered immediately after calving and 24 h later, n = 17); (3) delayed oral Ca supplementation (BOL-D; 43 g of oral Ca bolus administered 48 and 72 h after calving, n = 15); or (4) subcutaneous infusion (SQ; 500 mL of 23% Ca borogluconate infused subcutaneously once immediately after calving, n = 15).

Total Ca dynamics following subcutaneous Ca infusion and oral Ca bolus administration in multiparous Holstein cows have been previously reported (Blanc et al., 2014; Domino et al., 2017). These studies all showed numerical, and at times statistically significant, short-lived increases (maximum of 24 h) in serum Ca concentrations after oral Ca bolus administration with no evidence of a rebound hypocalcemia. Although Domino et al. (2017) reported a visual downward trend of serum Ca concentrations approximately 24 h after subcutaneous Ca infusion, none of these studies followed cows past 48 h after enrollment, so further effects on Ca concentration are unknown.

The prolonged reduction we found in tCa concentration over 168 h after administration of subcutaneous Ca is notable, as this appears to be an important time in early lactation when a reduction in tCa concentration is associated with an increase in disease events and reduced milk production (Neves et al., 2018; Couto Serrenho et al., 2021). Interestingly, Domino et al. (2017) showed in a large field trial that administration of 500 mL of 23% Ca borogluconate, the same supplementation provided in the current study, had no effect on disease events in the first 60 DIM or average weekly milk yield for the first 10 wk of lactation. Similar findings by Amanlou et al. (2016) and Miltenburg et al. (2016) showed transient 12 to 24 h increases in blood Ca concentration after subcutaneous Ca administration but reported no change in incidence of negative health outcomes or milk production assessed, on average, at 30 and 90 DIM, respectively, in studies randomizing approximately 100 to 500 cows per supplementation group. Thus, it is possible that an iatrogenic reduction in tCa during this period, alone, is not enough to increase the risk of adverse events, and that perhaps the presence of reduced tCa seen at this time of lactation in some cows is associated with another disease process, reduced DMI, or both. We hope future studies will investigate this relationship further.

Delaying Ca bolus administration to 48 and 72 h after calving did not affect tCa concentrations differently from conventional supplementation at calving and 24 h later. The tCa of either oral Ca supplementation group also did not differ from the CON group at any time point across 168 h after enrollment except at 120 h when the tCa of cows in the BOL-C group exceeded that of CON cows. Given other results surrounding comparison of the BOL-C and CON cows, it is possible this finding at 120 h is a type I error. Additional differences in tCa concentrations might exist among supplementation groups across time; however, our study sample size was not designed to detect differences in tCa concentrations at individual time points.

Our results suggest a possible difference in parity group among supplementation groups (*P* = 0.09). The CON group contained n = 3 and n = 12 cows in parity groups 2 and ≥3, respectively, with the BOL-C group including n = 4 and n = 13, the BOL-D group including n = 9 and n = 6, and the SQ group including n = 4 and n = 11 cows in each of the respective parity groups. In a study with a small sample size like ours, the potential impact of parity on the tCa outcome can be large given the natural Ca dynamics of older cows (Goff, 2014). This difference in group assignment is the result of unfortunate randomization differences between supplementation groups, and researchers should consider randomizing within parity group for future studies.

Contrary to our hypothesis, PTH concentrations did not differ between supplementation groups, suggesting that PTH responses to supplemental Ca, in the forms, dose, and routes of administration used in our study, are not solely responsible for the tCa dynamics we observed. Serum 0 h PTH did not differ among supplementation groups (*P* = 0.48) with group-level mean ± standard deviation concentrations of CON = 107.4 ± 50.0 pmol/L, BOL-C = 95.1 ± 34.0 pmol/L, BOL-D = 120.3 ± 63.7 pmol/L, and SQ = 103.2 ± 26.4 pmol/L. Modeled mean serum PTH concentrations over 168 h after enrollment for each supplementation group are in Table 1, and patterns of PTH over the study period are in Figure 1B. Parathyroid hormone concentrations in our study were relatively consistent across time but noticeably increased from calving to 8 h into lactation, likely a result of the immediate harvest of colostrum and initiation of milk production. Serum PTH did not differ over time among supplementation groups (*P* = 0.33) when controlling for 0 h PTH (*P* < 0.001) and parity group (*P* < 0.001). Concentrations of PTH over 168 h were greater for parity 2 cows at 167.0 pmol/L (95% CI = 160.5 to 177.4) compared with parity ≥3 cows at 148.0 pmol/L (95% CI = 142.3 to 153.7).

Whereas we calculated our study sample size to detect a difference in PTH concentration between groups at 96 h after calving, a biologically important time for Ca dynamics (McArt and Neves, 2020), it is possible this sample size was not large enough to detect a difference in other time points within the 168-h study period. In addition, our sampling in 8-h intervals might have been too infrequent to capture true changes in PTH. Other explanations for this lack of difference in PTH between groups include the possibility that tCa did not reach a high enough concentration after Ca administration to initiate the cascade of events that would reduce PTH signaling, or that calcitonin or other regulators of tCa concentration play a larger role in Ca homeostasis during the early-lactation period than PTH (Rodríguez et al., 2016; Connelly et al., 2021). Additionally, the analytical sensitivity of the assay might not have been precise enough to detect changes in PTH at these concentrations. There was also a large variation in PTH response between cows in our study, which might have prevented us from finding a true difference, if a difference did exist.

Although serum tCa did not differ between cows that received conventional or delayed oral Ca bolus supplementation at calving and cows that received no supplemental Ca, subcutaneous infusion of Ca at calving reduced serum tCa for a substantial period between 32 and 64 h postsupplementation. However, as PTH concentrations did not differ among groups across 168 h postpartum, the mechanism by which tCa was reduced remains unclear, and further investigation into the dynamics of Ca regulation mediators is needed to fully understand the impact that prophylactic Ca supplementation has on Ca dynamics during the early-lactation period.
